# Characterization of Platinum Nanoparticles Deposited on Functionalized Graphene Sheets

**DOI:** 10.3390/ma8095318

**Published:** 2015-09-21

**Authors:** Yu-Chun Chiang, Chia-Chun Liang, Chun-Ping Chung

**Affiliations:** Department of Mechanical Engineering, Yuan Ze University, 135 Yuan-Tung Rd., Chung-Li, Taoyuan 32003, Taiwan; E-Mails: s1000919@mail.yzu.edu.tw (C.-C.L.); s1000918@mail.yzu.edu.tw (C.-P.C.)

**Keywords:** platinum, graphene, functionalization, characterization, electrochemical activity

## Abstract

Due to its special electronic and ballistic transport properties, graphene has attracted much interest from researchers. In this study, platinum (Pt) nanoparticles were deposited on oxidized graphene sheets (cG). The graphene sheets were applied to overcome the corrosion problems of carbon black at operating conditions of proton exchange membrane fuel cells. To enhance the interfacial interactions between the graphene sheets and the Pt nanoparticles, the oxygen-containing functional groups were introduced onto the surface of graphene sheets. The results showed the Pt nanoparticles were uniformly dispersed on the surface of graphene sheets with a mean Pt particle size of 2.08 nm. The Pt nanoparticles deposited on graphene sheets exhibited better crystallinity and higher oxygen resistance. The metal Pt was the predominant Pt chemical state on Pt/cG (60.4%). The results from the cyclic voltammetry analysis showed the value of the electrochemical surface area (ECSA) was 88 m^2^/g (Pt/cG), much higher than that of Pt/C (46 m^2^/g). The long-term test illustrated the degradation in ECSA exhibited the order of Pt/C (33%) > Pt/cG (7%). The values of the utilization efficiency were calculated to be 64% for Pt/cG and 32% for Pt/C.

## 1. Introduction

Carbon materials are widely used as the supports for deposition of precious metal nanoparticles in heterogeneous and electrochemical catalysis [1,2]. The performance and cost efficiency of these catalyst materials are highly dependent on the dispersion and stability of the catalytic nanoparticles. Platinum (Pt) nanoparticles supported on carbon black (Pt/C) are currently considered the most promising electrocatalyst on proton exchange membrane fuel cells (PEMFCs). Respecting the fact that carbon blacks suffer serious corrosion problems under normal operating conditions, several nanostructured carbon materials have been investigated as Pt catalyst supports.

Herein, graphene, a monolayer of carbon atoms in a crystal lattices and the basal plane of graphite composed of a honeycomb arrangement of carbon atoms, receives special interest as a catalyst support in PEMFCs due to its distinct electronic, mechanical and structural properties, such as high electrical and heat conductivity, high chemical stability and large surface-to-volume ratio (theoretical specific surface area of 2620 m^2^/g) [3,4]. However, the weak interactions between metal nanoparticles and carbon supports generally result in severe agglomeration, dissolution, reprecipitation or migration of metal particles or the diffusion of metal ionic species, leading to the degradation of electrochemical activity under long-term operations. To enhance the interfacial interactions between the carbon supports and the Pt nanoparticles, the modifications of carbon supports are essential to develop the performance of Pt nanoparticles. Introduction of oxygen-containing functional groups onto the surface of carbon supports is a commonly used method to enhance the attachment and eliminate the agglomeration of Pt nanoparticles.

Li *et al.* [4] prepared composite graphene nanosheets decorated with Pt nanoparticles. Pt nano clusters, consisted of small Pt nanoparticles with mean diameters of about 5–6 nm, were deposited on the basal planes and the edges of the graphene. The value of the electrochemical surface area (ECSA) for the Pt/graphene was 44.6 m^2^/g, higher than that of commercial Pt/C (30.1 m^2^/g). Kou *et al.* [5] studied the electrochemical activity of Pt nanoparticles supported on functionalized graphene sheets (FGS) by impregnation methods. The improved performance of Pt/FGS compared to Pt/C (E-TEK) can be attributed to the smaller particle size (average Pt size ~2 nm) and less aggregation of the Pt nanoparticles on FGS. It was also observed Pt-Ru/graphene nanosheets exhibited higher ECSA (47.9 m^2^/g, 1.7 times that of Pt-Ru/Vulcan) and methanol oxidation activity (0.113 mA g/m^2^, 1.4 times that of Pt-Ru/Vulcan) [6]. Seo *et al.* [7] found the values of ECSA for Pd/graphene were 54.9 m^2^/g, higher than that of Pt/graphene (42.0 m^2^/g). In addition, some studies also investigated the effects of the spacers between metal nanoparticles and the graphene sheets, such as carbon nanotubes [8], carbon black [9], or indium tin oxide (ITO) [10].

It was observed that very small and highly dispersed PtRu nanoparticles decorated on a graphene support using simple surfactant-free synthesis processes possessed excellent activity and stability compared with conventional Pt/C (E-TEK) for the electrooxidation of biomass-derived glycerol [11]. The sulfhydryl groups uniformly attached on the graphene nanosheets were used to assemble gold nanoparticles. It was found that the gold particles (in the range of 3–5 nm) were highly scattered on the surface of the functionalized graphene sheets and this hybrid showed high electrochemical activity toward the oxygen reduction reaction and high stability in alkaline media [12]. Cobalt phosphate has also been prepared on porous graphene film by a convenient charge-controlled electrodeposition method, which served as an efficient oxygen evolving catalyst [13]. This graphene-supported catalyst was of high catalytic activity and stability towards water oxidation, superseding those of unsupported ones.

In this study, Pt nanoparticles were deposited on citric acid-treated graphene sheets (denoted as cG) using a reverse micelle method. Pt nanoparticles supported on carbon black (Pt/C) is currently considered the most promising electrocatalyst on PEMFCs. However, the corrosion of carbon black under operating conditions restricted the applications. Therefore, the graphene was applied to overcome this problem. To enhance the interfacial interactions between the graphene sheets and the Pt nanoparticles, the surface oxides were introduced onto the surface of graphene sheets. The products were characterized by high-resolution transmission electron microscopy (HRTEM), thermogravimetric analysis (TGA), X-ray diffraction (XRD), X-ray photoelectron spectroscopy (XPS) and Fourier transform infrared spectroscopy (FT-IR). The cyclic voltammetry (CV) technique was used to measure the ECSA, stability and utilization efficiency of the catalysts. One commercial Pt/C (Johnson Matthey) was used for comparison.

## 2. Experimental Section

### 2.1. Modification of Graphene Sheets

The commercial graphene sheets (denoted as GS) used in this study were provided by Legend Star International Co. (New Taipei City, Taiwan). The oxygen-containing functional groups were introduced onto the surface of the graphene sheets by treating them with citric acid (C_6_H_8_O_7_), as follows: 200 mg of graphene sheets were added to the citric acid aqueous solution (1.6 mM) and subjected to 15 min ultrasonic treatment, followed by vigorous stirring for 1 h using a magnetic stirrer. The ultrasonic treatment was conducted using an ultrasonic cleaner (LEO-1502, LEO Ultrasonic Co., New Taipei City, Taiwan) at a frequency of 46 kHz and a power of 150 W. The solvent was removed by filtering and the samples were heat-treated in a muffle furnace at 300 °C for 30 min. This product was denoted as cG.

### 2.2. Synthesis of Pt/cG

**Figure 1:**
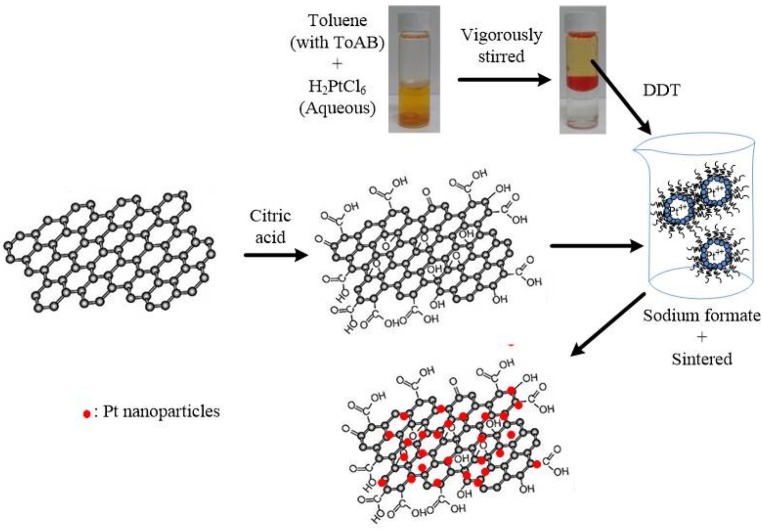
Schematic representation of surface modification of graphene sheets and the deposition of Pt nanoparticles.

The deposition of Pt particles on the oxidized graphene sheets was performed using the colloidal method [14], involving a two-phase transfer of PtCl_6_^2−^, followed by reduction in the presence of 1-dodecanethiol (DDT, C_12_H_25_SH, 98%) (Sigma-Aldrich, Steinheim, Germany). Hexachloroplatinic acid (H_2_PtCl_6_·6H_2_O) solution (3 mL of 0.086 M in deionized water) (Alfa Aesar, Heysham, England) was mixed with tetraoctylammonium bromide (ToAB, N(C_8_H_17_)_4_Br) (Sigma-Aldrich, Steinheim, Germany) solution (4 mL of 0.18 M in toluene) and vigorously stirred for about 30 min at room temperature. During this process, PtCl_6_^2−^ ions are transferred from the aqueous solution to the organic layer (toluene), using ToAB as the phase-transfer catalyst. The orange-colored organic layer was extracted and 450 mg of DDT (capping agent) was added and stirred for 30 min. 200 mg cG was then added with constant stirring for 1 h. 10 mL of 0.25 M sodium formate (HCOONa) aqueous solution was added drop-wise at 60 °C, to reduce the Pt ion. The solid product was filtered and rinsed with ethanol to remove excess DDT and copious amount of warm deionized water was used to remove the remaining sodium formate. The resulted Pt/cG products were dried at 100 °C for 3 h and further heat-treated at 800 °C for 2 h in a tubular furnace in an argon atmosphere. Figure 1 illustrated the schematic representation of surface modification of graphene sheets and the deposition of Pt nanoparticles. For comparison, the commercial Pt/C with a Pt content of 20 wt % (Johnson Matthey, denoted as JM) was purchased.

### 2.3. Material Characterizations

HRTEM images were obtained using a transmission electron microscope (Tecnai G2, 200 kV) (Philips/FEI, Eindhoven, the Netherlands), to determine the interior microstructure of the graphene sheets and the morphology and size distribution of the Pt particles deposited on the graphene sheets. The oxidation resistance of the samples was determined in flowing air (60 cm^3^/min) with a heating rate of 10 °C/min, using a thermogravimetric analyzer (Dynamic TGA Q500 in TA Instrument 5100) (New Castle, DE, USA) to measure any changes in the weight of the sample as a function of temperature (TGA plot) and the rate of weight loss *versus* temperature (differential thermogravimetry, DTG, plot). The residual mass was used to estimate the Pt content deposited on the surface of the graphene sheets. The XRD patterns were taken with an X-ray powder diffractometer (Rigaku TTRAX III, Tokyo, Japan) to give detailed information about the crystallographic structure of the materials. The radiation used was Cu Kα with a wavelength of 0.15418 nm, a voltage of 30 kV and a current of 20 mA. The value of 2-theta (2θ) ranges from 10° to 90°, where θ is the diffraction angle, with a scanning speed of 4°/min. XPS was used to determine the number and type of functional groups present on the surface of the samples, using a spectrophotometer (PerkinElmer PHI 1600, Waltham, MA, USA). A twin anode Mg X-ray source (hν = 1253.6 eV) was used, at a voltage of 15 kV and a power of 400 W. For calibration purposes, the C1s electron binding energy that corresponds to graphitic carbon was set at 284.6 eV. A nonlinear least squares curve-fitting program (XPSPEAK software, Version 4.1) was used for deconvolution of the XPS spectra. The FT-IR spectra were recorded using a Fourier transform infrared spectroscope (PerkinElemer Spectrum 100, Waltham, MA, USA) with KBr pellets in the 4000–400 cm^−1^ region to characterize the functional groups on the samples.

### 2.4. Measurement of Electrochemical Activity

A CHI 613C electrochemical workstation (CH Instruments, Austin, TX, USA) was employed for the electrochemical study of carbon-supported Pt samples. A three-electrode electrochemical cell was constructed for CV measurements, through which the ECSA of the Pt nanoparticles was determined. The working electrode was a thin layer of Nafion^®^-impregnated catalyst sample, cast on a vitreous carbon disk of 5 mm in diameter embedded in a Teflon cylinder. A homogeneous ink composed of electrocatalyst, Nafion^®^ ionomer (5 wt %, DuPont), and isopropanol, where the Nafion^®^ content in the thin-film electrocatalyst layer was 25 wt %. A Pt wire and a saturated calomel electrode (SCE) were used as the counter and reference electrodes, respectively. The measurements of CV were conducted at room temperature using 0.5 M H_2_SO_4_ as the electrolyte solution at a scan rate of 20 mV/s from −0.2 to 1.0 V *vs.* SCE.

## 3. Results and Discussion

**Figure 2:**
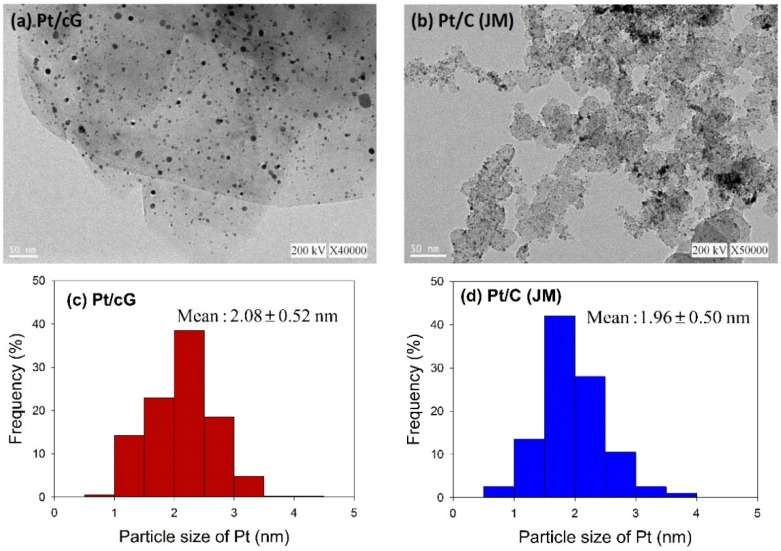
HRTEM images (**a**) and (**b**) and Pt particle size distribution (**c**) and (**d**) of the samples.

The morphology of the prepared catalyst was observed using HRTEM. Figure 2 shows the HRTEM images and the distribution of the Pt particle sizes for Pt/cG and Pt/C (JM). The flake-like shapes of graphene sheets and the wrinkles on the graphene sheets were clearly observed in Figure 2a. Pt nanoparticles were uniformly dispersed on the surface of the graphene sheets (Figure 2a) and carbon black (Figure 2b) and the sizes ranged between 0 and 5 nm with some nano clusters. The mean sizes of the Pt particles were 2.08 ± 0.52 nm (Pt/cG), similar to the study of Kim *et al.* [11] and slightly greater than Pt/C (JM) (1.96 ± 0.50 nm). As seen from the data, the Pt nanoparticles deposited on the graphene sheets had a similar particle size and distribution to that of Pt/C, and both had a high degree of uniformity.

**Figure 3:**
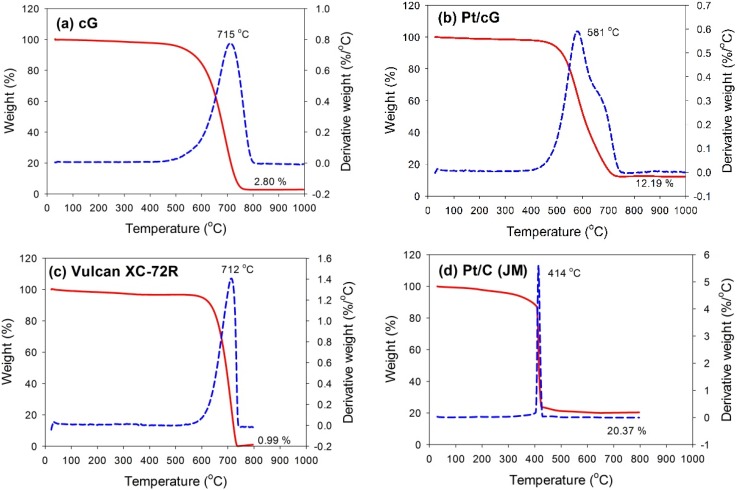
TGA profiles of the samples. (**a**) cG; (**b**) Pt/cG; (**c**) Vulcan XC-72R; (**d**) Pt/C (JM).

The TGA and DTG profiles measured in flowing air for all samples are shown in Figure 3. The samples were heated at a rate of 10 °C/min in an air flow of 60 sccm. The temperatures for the maximum rate of weight loss (or oxidation) for cG, Pt/cG and Pt/C were 715, 581 and 414 °C (Figure 3a,b,d). The maximum rate of weight loss for cG occurred at a temperature higher than that of the carbon nanotubes in the literature. Following deposition with Pt nanoparticles, the maximum rate of weight loss slightly decreased. Nevertheless, the thermal stability or the resistance to oxidation of Pt/cG was still higher than Pt/C (JM). The TGA and DTG profiles of Vulcan XC-72R (the support of Pt/C (JM)) were provided in Figure 3c, whose thermal stability was similar to cG. However, the deposition of Pt nanoparticles on the Vulcan carbon significantly decreased its resistance to oxidation. Figure 3b depicted two peaks for the DTG profile, which was attributed to the reason that different resistance to oxidation existed on the Pt/cG samples. The graphene sample used in this study was multi-layered. The Pt nanoparticles were mainly distributed on the outermost sheets where the thermal stability would be inferior to the internal sheets without Pt deposition. Thus, there were two peaks on the DTG profile. The profile for the TGA weight loss of Pt/cG showed the residue was 12.19 wt %. This datum, combined with the metal catalyst residue in cG, was used to calculate the Pt content, which was about 10 wt %.

**Figure 4:**
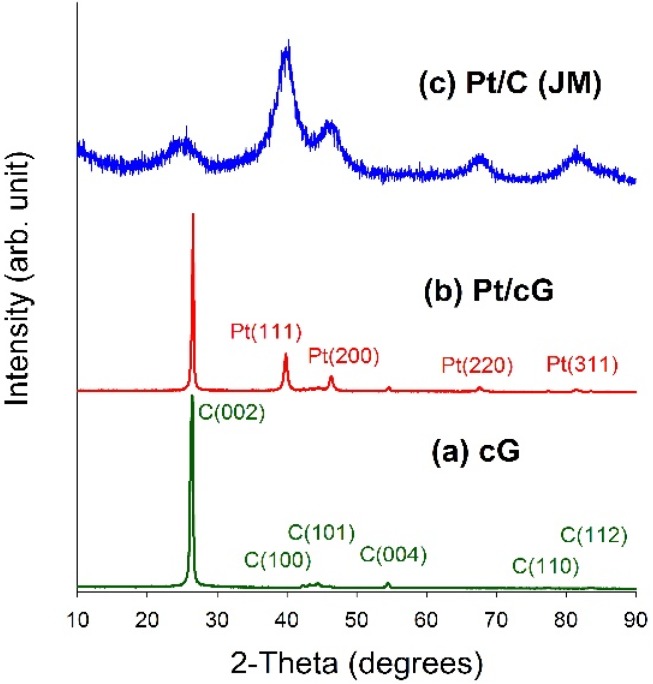
XRD patterns of the samples. (**a**) cG; (**b**) Pt/cG; (**c**) Pt/C (JM).

Figure 4 displayed the XRD patterns for all samples. As seen from Figure 4a,b, a high degree of crystallinity for cG and Pt/cG was observed. The diffraction peaks at 2θ angles of 26.4°–83.2° in the XRD pattern of oxidized graphene sheets can be assigned to hexagonal crystalline graphite (JCPDS No. 41-1487). The peaks at 2θ angles of 26.4°, 42.2°, 44.4°, 54.5°, 77.2° and 83.2° could be indexed as the reflections from the C(002), C(100), C(101), C(004), C(110) and C(112) planes. Pt/cG exhibited strong diffraction peaks at 2 θ values of 39.8°, 46.2°, 67.5° and 81.3°, which could be indexed as the reflections from the Pt(111), Pt(200), Pt(220) and Pt(311) facets of the face-centered cubic structures of Pt crystal (JCPDS No. 4-802). The diffraction peaks for Pt/C (JM) were broader than those for Pt/cG, implying the average size of the Pt particles on Pt/cG was greater than that on Pt/C, consistent with the conclusions from the HRTEM images. In summary, the XRD results show the Pt precursor is reduced to the metallic state using the colloidal method.

**Figure 5:**
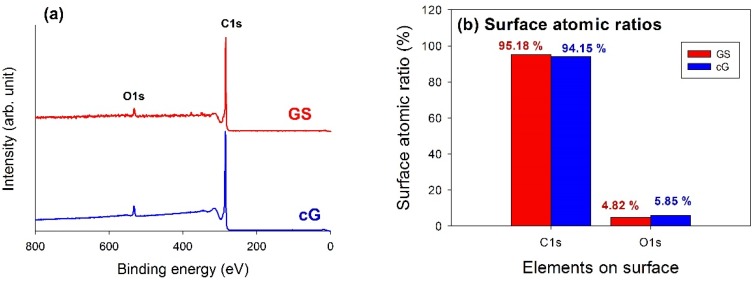

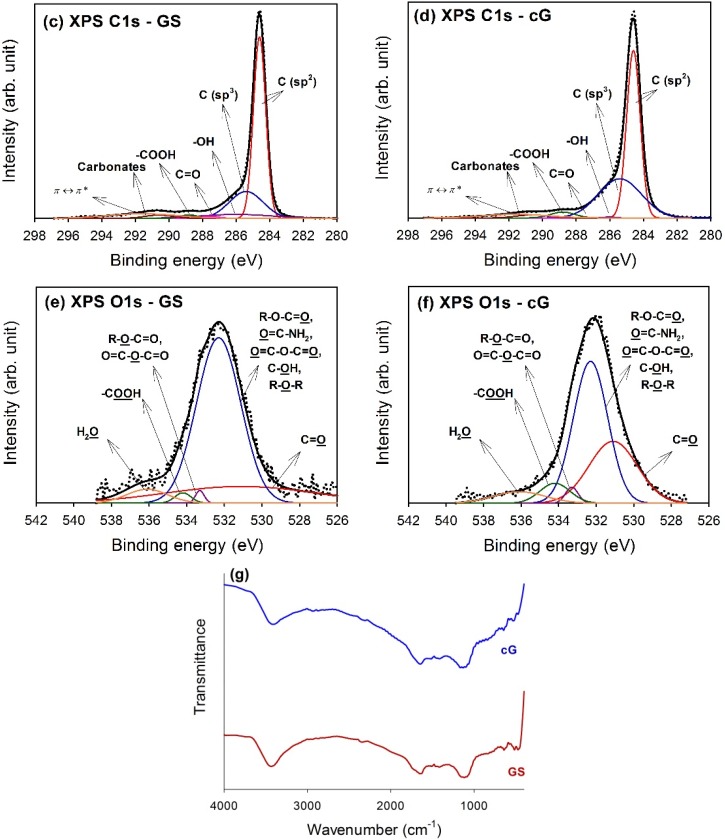
Chemical states of GS and cG. (**a**) XPS survey scan spectra; (**b**) surface atomic ratios; (**c**) and (**d**) curve fitting of high resolution XPS C1s spectra; (**e**) and (**f**) curve fitting of high resolution XPS O1s spectra; (**g**) FT-IR spectra.

XPS analysis was used to elucidate the surface compositions and the oxidation states of the materials. The XPS survey scan spectra of the samples revealed the compositions of the most external surface. Data from the XPS survey spectra of the GS and cG (Figure 5a) indicated the major peaks in the spectra were due to the C1s and O1s photoelectrons. Figure 5b illustrated the elemental compositions (measured in atomic ratio, at.%) on the surface or over the sampling depth of several atomic layers from the surface. The oxidation of weak citric acid led to a slight increase in the percentage of oxygen.

The high-resolution XPS spectra of the C1s region for GS and cG were illustrated in Figure 5c,d. Carbon atoms differed in their binding energies depending on whether they are linked to one O atom by a single bond, a double bond, or two oxygen atoms [15]. Deconvolution of the C1s spectra gave seven individual component groups. Table 1 summarized the calculated percentages of graphitic and functional carbon atoms, where the values were given in at.% of total intensity. Besides graphitic carbon, phenolic groups were the most predominant functionalities on the surface of the GS. The percentage of carbon atoms in aliphatic structures (C(sp^3^)) obviously increased once the citric acid oxidation was applied. Moreover, the content of the –COOH groups was also significantly enhanced and the surface became much more active due to the decrease in the π-π* transitions in aromatic rings.

**Table 1:** Results of the fits of the XPS C1s region, values given in at.% of total intensity.

Sample	Binding Energy (eV)						
	284.6	285.4	286.0	287.6	288.8	290.6	291.6
	Carbon Atoms in Polyaromatic Structures (C(sp^2^))	Carbon Atoms in Aliphatic Structures (C(sp^3^))	–OH	C=O	–COOH	Carbonates	π-π*
GS	56.56	22.88	7.31	1.08	1.54	2.41	8.22
cG	51.25	37.86	0.25	0.10	3.16	2.17	5.21

To gain a better understanding of the results noted above, high-resolution O1s spectra of GS and cG were illustrated in Figure 5e,f, and four different O functionalities as well as a contribution from chemisorbed water were identified, as reported in the studies by Zielke *et al.* [16,17]. The calculated percentages of functional O atoms were shown in Table 2. As seen from the data, the citric acid oxidation resulted in the decrease of the –OH groups but the increase of the –COOH groups on graphene sheets, consistent with the result for the high-resolution C1s region. Specifically, except for –OH groups, the percentages of other O functionalities increased after oxidation. The FT-IR spectra in Figure 5g provided another piece of evidence for the above discussion, which was in agreement with the literature [18,19]. The bands centered at ~3470 and ~1400 cm^−1^ were attributed to deformation of the –OH bond and CO–H groups. The stretching vibrations of the carbonyl or carboxyl groups and the C–O bond were observed at ~1670 and 1100 cm^−1^, respectively.

**Table 2:** Results of the fits of the XPS O1s region, values given in at.% of total intensity.

Sample	Binding Energy (eV)				
	531.1	532.3	533.3	534.2	536.1
	C=O	R-O-C=O, O=C-NH_2_, O=C-O-C=O, C-OH, R-O-R	R-O-C=O, O=C-O-C=O	C-OOH	H_2_O
GS	23.55	68.53	1.02	1.57	5.33
cG	32.24	52.54	2.20	6.53	6.49

**Figure 6:**
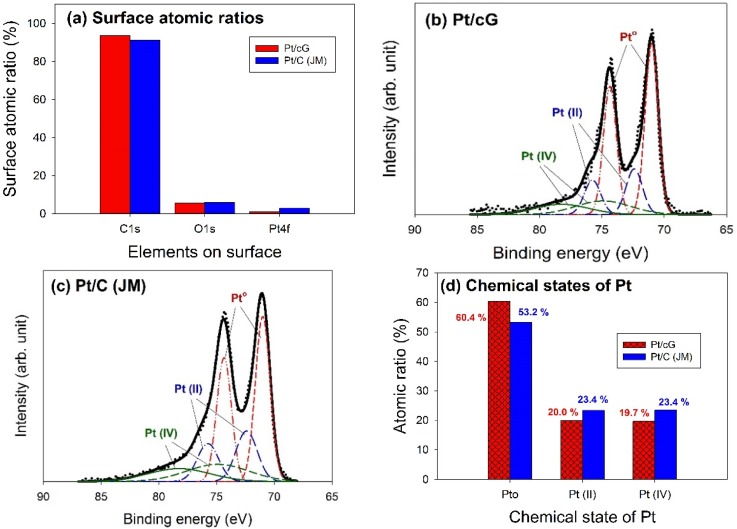
Curve fitting of high-resolution XPS Pt4f spectra for the samples. (**a**) Surface atomic ratios; (**b**) Pt/cG; (**c**) Pt/C (JM); (**d**) Chemical states of Pt.

The major peaks observed in the survey scan spectra of Pt/cG and Pt/C (JM) were due to the C1s, O1s, and Pt4f photoelectrons. Figure 6a showed the atomic ratios of C1s, O1s, and Pt4f for Pt/cG and Pt/C (JM), where the ratios were similar for both samples. The most abundant element on the surface was carbon. The content of oxygen atoms implied the surface was not hydrophilic enough. Moreover, the Pt contents from XPS agreed with those from the TGA profiles.

The deconvolution of the XPS spectra over the Pt4f region, shown in Figure 6b,c, showed different states of oxidation for each of the carbon supported Pt samples, consisting of three couples of doublets. The most intense doublet, at 71.0 and 74.35 eV, represents a zero-valence metallic Pt (Pt°), the doublet at 72.4 and 75.75 eV is attributed to the presence of an amorphous Pt(II) species, such as PtO or Pt(OH)_2_ [20], and the broader doublet at 74.9 and 78.25 eV is assigned to the Pt(IV) species. Figure 6d summarized the calculated percentages of the Pt species in different chemical states. The data showed most of the Pt was in the zero-valence metallic state (>50%). The percentages of Pt°, Pt(II), and Pt(IV) in Pt/cG were 60.4, 20.0 and 19.7 at.% and in Pt/C (JM) were 53.2%, 23.4% and 23.4%. Pt/cG had higher Pt°, but less Pt(II) and Pt(IV), indicating the Pt nanoparticles deposited on graphene sheets were reduced much more effectively, compared to those on carbon blacks.

**Figure 7:**
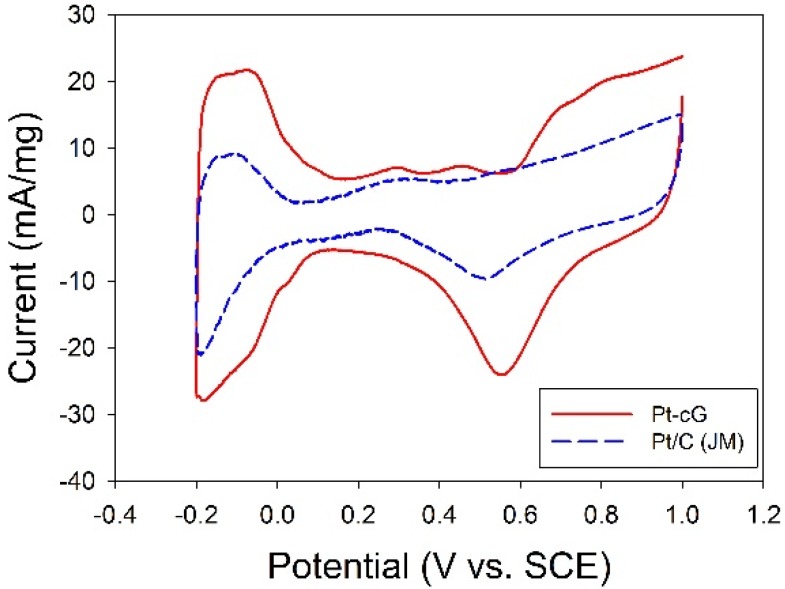
Cyclic voltammograms and changes in ECSA for all samples.

The cyclic voltammogram for the Pt/cG was displayed in Figure 7 and the cyclic voltammogram of the Pt/C (JM) was also presented for comparison. The CV curves show three characteristic potential regions: the hydrogen adsorption/desorption region (−0.2 to 0.1 V), double layer plateau region (0.1 to 0.5 V) and the formation and reduction of surface Pt oxides (0.5 to 1.0 V). All voltammograms display a well-defined hydrogen adsorption/desorption region from −0.2 to 0.1 V *vs.* SCE. Compared to that of the Pt/C, the Pt nanoparticles deposited on the graphene sheets possessed a larger loop, especially a wide double layer region, indicating the system’s greater ability to store electric charges. The ECSA is a very significant parameter indicating the intrinsic electrocatalytic activity of a Pt catalyst. The values of the ECSA for Pt/cG and Pt/C were calculated to be 88 and 46 m^2^/g Pt, respectively, based on a monolayer hydrogen adsorption charge of 0.21 mC/cm^2^ on polycrystalline Pt. A higher ECSA of the Pt/cG could be attributed to the small Pt nanoparticles and the efficient formation of zero-valence metallic Pt such that the fuel gases could attach to the redox sites on Pt nanoparticles. Note that the Pt/cG required much more activation time to develop its electrochemical activity.

To understand the stability of the electrochemical activity of the graphene sheets supported Pt nanoparticles, the long-term operation of CV analysis up to 600 cycles was conducted under the same conditions (at a scan rate of 20 mV/s from −0.2 to 1.0 V *vs.* SCE) and the values of ECSA were evaluated. The dependence of the values in ECSA on the number of cycles (Table 3) showed degradation in ECSA due to dissolution/reprecipitation or migration of Pt particles or the diffusion of Pt ionic species [21] presented in the order of Pt/C (33%) > Pt/cG (7%). The degradation of electrochemical activity of Pt/cG was negligible within the experimental error threshold. It was believed the graphene corrosion was unexpected and the oxidation of the Pt surface was slowed down. Evidently, the loss in ECSA up to 600 cycles for Pt/cG was only 21% of that associated with commercial Pt/C (JM) catalysts, implying the electrostatic attractive force between Pt colloids and oxidized graphene sheets greatly surpassed that on the Pt/C (JM) catalysts. In addition, the higher electrochemical activity of Pt/cG was attributable to the 2D nanostructure, large surface area, and good conductivity of cG [8]. The multi-layer structure of graphene sheets also possesses a higher porosity leading to a better mass transport of electrolytes and reactants. The functional groups on graphene sheets may lead to a strong metal-support interaction, resulting in resistance of Pt to agglomeration and sintering [5], and therefore enhanced durability.

**Table 3:** Calculated ECSA (m^2^/g) and activity degradation (%) of the samples.

Sample	Parameter	30th	100th	200th	300th	400th	500th	600th
Pt/cG	ECSA (m^2^/g)	82	88	87	85	84	84	82
	Degradation (%)	-	-	2	3	5	5	7
Pt/C (JM)	ECSA (m^2^/g)	46	40	34	33	33	33	31
	Degradation (%)	-	13	26	28	29	28	33

In addition, the Pt utilization efficiency (η) is also the essential parameter to describe the catalyst performance, which was calculated by dividing ECSA by the chemical surface area (CSA) [22]. The CSA is defined as 6/ρd, where ρ is the density of Pt (=21.09 g/cm^3^) and d is mean diameter of Pt nanoparticles (nm). The values of the η were calculated to be 64% for Pt/cG and 32% for Pt/C. The results implied oxidized graphene sheets were an effective support for Pt depositions.

## 4. Conclusions

The results showed the Pt nanoparticles on Pt/cG were uniformly dispersed on the surface of graphene sheets by the reverse micelle method, where the mean size of the Pt particles was 2.08 ± 0.52 nm, and the average size of the Pt particles on Pt/C (JM) was 1.96 ± 0.50 nm. The Pt nanoparticles deposited on graphene sheets exhibited better crystallinity and the sample had a higher oxygen resistance. The metal Pt was the predominant Pt chemical state on Pt/cG (60.4%) and Pt/C (53.2%). The results from CV analysis showed the values of ECSA were 88 m^2^/g (Pt/cG) and 46 m^2^/g (Pt/C), respectively. The long-term test illustrated the degradation in ECSA exhibited the order of Pt/C (33%) > Pt/cG (7%). The values of the η were calculated to be 64% for Pt/cG and 32% for Pt/C. Therefore, Pt nanoparticles deposited on functionalized graphene sheets should produce a promising electrocatalyst.
